# Vitamin B_1_ in marine sediments: pore water concentration gradient drives benthic flux with potential biological implications

**DOI:** 10.3389/fmicb.2015.00434

**Published:** 2015-05-12

**Authors:** Danielle R. Monteverde, Laura Gómez-Consarnau, Lynda Cutter, Lauren Chong, William Berelson, Sergio A. Sañudo-Wilhelmy

**Affiliations:** ^1^Department of Earth Sciences, University of Southern CaliforniaLos Angeles, CA, USA; ^2^Department of Biological Sciences, University of Southern CaliforniaLos Angeles, CA, USA

**Keywords:** vitamin B_1_, thiamin, coenzyme, sediment, flux, auxotroph

## Abstract

Vitamin B_1_, or thiamin, can limit primary productivity in marine environments, however the major marine environmental sources of this essential coenzyme remain largely unknown. Vitamin B_1_ can only be produced by organisms that possess its complete synthesis pathway, while other organisms meet their cellular B_1_ quota by scavenging the coenzyme from exogenous sources. Due to high bacterial cell density and diversity, marine sediments could represent some of the highest concentrations of putative B_1_ producers, yet these environments have received little attention as a possible source of B_1_ to the overlying water column. Here we report the first dissolved pore water profiles of B_1_ measured in cores collected in two consecutive years from Santa Monica Basin, CA. Vitamin B_1_ concentrations were fairly consistent between the two years ranging from 30 pM up to 770 pM. A consistent maximum at ~5 cm sediment depth covaried with dissolved concentrations of iron. Pore water concentrations were higher than water column levels and represented some of the highest known environmental concentrations of B_1_ measured to date, (over two times higher than maximum water column concentrations) suggesting increased rates of cellular production and release within the sediments. A one dimensional diffusion-transport model applied to the B_1_ profile was used to estimate a diffusive benthic flux of ~0.7 nmol m^−2^ d^−1^. This is an estimated flux across the sediment-water interface in a deep sea basin; if similar magnitude B-vitamin fluxes occur in shallow coastal waters, benthic input could prove to be a significant B_1_-source to the water column and may play an important role in supplying this organic growth factor to auxotrophic primary producers.

## Introduction

Vitamin B_1_ (thiamin) is a soluble, biotically synthesized, heterocyclic sulfur, and nitrogen-containing catalyst required in trace amounts by all organisms (Jurgenson et al., 2009). It is primarily used as a coenzyme in forming and breaking C-C bonds and is required in central metabolic processes including the pentose-phosphate pathway and tricarboxylic acid cycle as well as in acetolactate synthase utilized in the synthesis of branched-chain amino acids (Frank et al., 2007). This vitamin was originally identified as the molecule in rice husks which cures the human disease beriberi, caused by vitamin B_1_ deficiency, a discovery which was recognized with the 1929 Nobel Prize in Physiology and Medicine (Eijkman, 1990). In the 1950s and 60s it was discovered that some species of marine phytoplankton are unable to synthesize B_1_ *de novo* (B_1_ auxotrophs) and instead must acquire the coenzyme from an exogenous source (Droop, 1957; Provasoli, 1958; Carlucci and Silbernagel, 1969). This included many of the major marine primary producers (Croft et al., 2006; Bertrand and Allen, 2012; Sañudo-Wilhelmy et al., 2014) as well as some ubiquitous marine bacteria (Giovannoni et al., 2005) and picoeukaryotic algae (Paerl et al., 2015). However, not every marine microbe (including both bacterioplankton and phytoplankton) requires an exogenous source of B_1_; many microbes possess the full metabolic pathway needed to synthesize B_1_ (Sañudo-Wilhelmy et al., 2014). Interestingly, B_1_ auxotrophs do not appear to be related phylogenetically indicating that the loss of synthesis capability likely occurred multiple times (Helliwell et al., 2013). Additionally, it was discovered that B_1_ synthesizers (or B_1_ prototrophs) are able to self-regulate physiological concentrations within the cell via the use of a riboswitch (Croft et al., 2007). Recent work has revealed an additional layer of complexity regarding B_1_ proto- and auxotroph dynamics, in that some species may only possess part of the synthesis pathway and can scavenge B_1_ and/or its precursor moieties (4-amino-5-hydroxymethyl-2-methylpyrimidine or 4-methyl-5-β-hydroxyethylthiazole) in order to obtain the complete and active form of this vitamin (Jurgenson et al., 2009). Such organisms include climatologically relevant eukaryotic species such as *Emiliani huxleyi* (McRose et al., 2014) as well as environmentally abundant bacteria in the SAR11 clade (Carini et al., 2014). Field studies investigating dissolved B-vitamins in marine systems have shown that phytoplankton species succession and biomass production are influenced by the availability of vitamins B_12_ (cobalamin) and B_1_ (Sañudo-Wilhelmy et al., 2006; Panzeca et al., 2009; Koch et al., 2011; Bertrand et al., 2012). Additionally, it has been found that large regions of the ocean appear to be depleted in B_1_ as well as other B-vitamins (Sañudo-Wilhelmy et al., 2012). Despite this, the sources of this organic growth factor have not been clearly identified and research in the area has mainly focused on B-vitamin production within the water column (e.g., Koch et al., 2012).

Marine sediments pose a potentially significant source for B_1_ since sediments contain some of the highest cellular densities and diversity of any environment on Earth (where cellular abundance can reach as high as 10^9^ cells/cm^3^; Kallmeyer et al., 2012). Pioneering work in the 1950s and 1960s revealed that marine sediments may serve as a source of some B-vitamins including vitamin B_1_ (Burkholder and Burkholder, 1958; Burkholder and Lewis, 1968). Based on our survey of required synthesis genes in whole genome sequenced sediment isolates (see Supplementary Material Table S1), marine sediments include many potential B_1_ prototrophs. However, the majority of sediment microbes have remained uncultured (Eilers et al., 2000), and the dynamics of B_1_ production and extracellular release remain largely unexplored. Thus, measuring dissolved B_1_ in sediment pore waters is essential to determine if the marine sediment community as a whole serves as a source of this critically required vitamin. In comparison to the many decades of study on trace metal and inorganic nutrient requirements (e.g., Fe and NO^−^_3_), vitamins have received substantially less attention as a limiting agent to productivity. This is due in part to difficulties encountered measuring a labile molecule found in trace amounts (femto to pico molar concentrations) via the classic bioassay techniques or with the more recently developed liquid chromatography-mass spectrometry (LC/MS) techniques which can provide compound-specific information (Carlucci and Silbernagel, 1966; Okbamichael and Sañudo-Wilhelmy, 2005). As a result, the existing published environmental measurements of dissolved B_1_ are almost entirely focused on the water column with little attention given to marine sediments and their pore waters. As such, we pose the following targeted research questions: (1) What are the sediment pore water B_1_ concentrations? (2) Is there a flux of B_1_ from sediments to the overlying water? (3) How relevant are those concentrations and fluxes to biological communities in the water column?

To address these questions, this study presents the first dissolved B_1_ concentration profiles in marine pore waters, collected from the California Borderlands in Santa Monica Basin (SMB), CA from two sampling years (2011 and 2012). Pore water concentrations were compared to water column concentrations collected at the same station (Sañudo-Wilhelmy et al., 2012). Finally a simple diffusion-transport model was applied to the B_1_ pore water concentrations in order to establish the first diffusive benthic flux estimates of B_1_ from marine sediments.

## Materials and methods

### Study site

SMB lies ~10 miles offshore from Los Angeles within the California Continental Borderlands region. The basin is steep-walled with a flat-bottom covering an area of ~1800 km^2^ with a basin floor at ~910 m and a sill at ~740 m isolating sub-sill waters from mixing with nearby basins; flushing events are estimated to occur every 1–8 years (Hammond et al., 1990; Berelson, 1991; Hickey, 1991; Berelson and Stott, 2003). Bottom waters and surface sediments are nearly but never completely anoxic (<10 μM oxygen) yet oxygen is undetectable within the first few millimeters of the sediment column (Shaw et al., 1990; Berelson et al., 2005). As a result of the low oxygen concentrations in bottom waters, the sediments are laminated with no evidence of infauna and minimal bioturbation indicating little to no advective mixing of pore waters (Jahnke, 1990; Christensen et al., 1994; Berelson et al., 2005; Tems et al., 2015). Previous studies indicate a ~5 cm-deep ferruginous/manganous zone defined by maximum concentrations of dissolved iron, and manganese (Jahnke, 1990; McManus et al., 1998; Prokopenko et al., 2011). Beneath this lies a zone with decreasing dissolved iron and manganese concentrations (Jahnke, 1990; McManus et al., 1998; Burdige and Komada, 2011). Flushing events, minor bioturbation, and changes in surface primary productivity may cause seasonal changes in shallow sediment (~0–5 cm) geochemical zonation by introducing increased concentrations of oxygen, nitrate, and/or particulate organic carbon (Berelson, 1991). Multiple studies have investigated SMB sediment accumulation rates and found that roughly 9–11% of surface water primary productivity is exported to the basin floor resulting in consistent hemipelagic-sourced sediments accumulating at ~16.0 ± 3 mg cm^−2^ y^−1^ (Huh et al., 1990; Christensen et al., 1994; Berelson and Stott, 2003). In nearby San Pedro Basin particle flux was found to be seasonal and SMB likely experiences similar seasonality in sediment input (Collins et al., 2011). Of the organic carbon that reaches the sediment floor, ~40% is buried and preserved while the rest is remineralized and escapes to the water column (Jahnke, 1990). SMB's sediments are characterized as a silty-clay with ~10% calcium carbonate content and ~4–6% organic carbon (Craven and Jahnke, 1992; Gorsline, 1992). Sediments follow a typical porosity profile starting around a porosity of 0.98 which exponentially decreases with depth to values of ~0.85 at 8 cm depth (Berelson et al., 2005; Komada et al., 2013).

### Core collection

**Figure 1:**
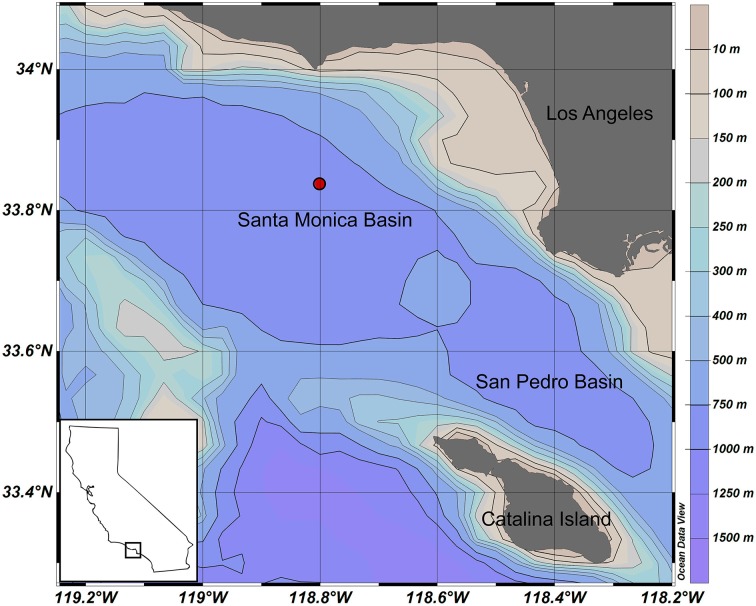
**Santa Monica Basin station location (33°48.76′ N, 118°46. 60′ W)**. This figure was generated using Ocean Data View (Schlitzer, R. Ocean Data View, http://odv.awi.de, 2015).

Sediment cores were collected from SMB (33°48.76′ N, 118°46.60′ W; Figure 1) far enough away from basin walls and on a small regional high to avoid turbidite sampling. Cruises occurred in January 2011 and March 2012, just prior/during the expected maximum particle flux but before any spring flushing. Cores of 25–45 cm length were collected with an Ocean Instruments (MC 400) multicorer (Barnett et al., 1984) containing 9.5 cm diameter core liners. Upon retrieval, cores were inspected for a well-preserved sediment-water interface, minimal overlying water turbidity, and a lack of bubbles in the sediment in order to minimize collection artifacts. All cores were stored on board ship in an ice bath protected from light until transport to the laboratory cold room for sampling ~9 h after retrieval. Cores were sampled at depths of 1, 3, 5, 7, 9, 11, 15, 20, 25, and 35 cm in 2011 and 1.5, 3.5, 5.5, 7.5, 11.5, 15.5, 19.5, 25.5, 31.5, and 39.5 cm in 2012 using Rhizon soil samplers (Rhizosphere Research Products) fitted with 0.2 μm pore size filters. Rhizons were inserted into pre-drilled holes in the core liner and pore water was collected on cm-scale resolution using plastic syringes (Norm-ject) which had been acid-cleaned and methanol-rinsed. Sample volume ranged from 5 to 30 mL. Samples were then passed through an acid-washed 0.2 μm polypropylene capsule filter and stored frozen in acid-cleaned and methanol-rinsed high-density polyethylene (HDPE) amber bottles until analysis. Samples were protected from light as much as possible throughout sample processing. The 2012 dissolved iron samples were collected and filtered in the same way as the vitamin samples. Samples were stored in trace metal-cleaned HDPE bottles and acidified with Optima grade hydrochloric acid to a pH <2 following standard trace clean techniques.

### Analytical methods

Vitamin B_1_ was measured according to the technique described previously (Sañudo-Wilhelmy et al., 2012). The technique involves a solid-phase extraction onto a C_18_ resin at pH 6.5 and 2.0 followed by elution with methanol, drying, and quantification using high-performance liquid chromatography/tandem triple quadrupole mass spectrometer (LC/MS) with an electrospray ionization interface. Reagent grade thiamin hydrochloride (≥99%) was obtained from Sigma-Aldrich and used as an external standard. Samples were triple injected into the LC/MS to confirm instrument stability. Because sample volume is so low replicate sample splits were not performed. Method analytical blanks were measured with Milli-Q water subjected to the same preconcentration and quantification steps resulting in 0 pM in 2011 and 3.7 pM in 2012, which was subtracted from all of the sample measurements. The detection limits of the technique were 11 pM in 2011 and 0.53 pM in 2012 defined as three times the standard deviation of the procedural blank for 2012 and three times the standard deviation of the y-intercept of the calibration curve divided by the slope following the method outlined by Snyder et al. (2010) since the procedural blank was equal to zero in 2011. The improvement in detection limits between years resulted from an optimization of the method by increasing the sample injection volume from 50 to 100 μL. Dissolved iron concentrations were quantified by ICP-MS using external calibration curves and an internal indium standard.

### Benthic flux model

We applied a one-dimensional diffusion-transport model following Fick's First Law to the B_1_ pore water concentration profile. The data (from 0 to 9 cm) was fit with a polynomial function:

$$\text{C}={\text{C}}_{0}+{\text{m}}_{1}x+{\text{m}}_{2}x^{2}$$

Where *x* is depth (cm), m_1_ and m_2_ are fitting parameters, and C_0_ is the concentration at the sediment water interface (SWI). The diffusive flux of B_1_ across the SWI was determined using Fick's First Law, applied to the derivative of the polynomial function fit evaluated at *x* = 0:

$$\text{J =}-\varphi^{3}{\text{D}}_{\text{o}}\left(\frac{dc}{dx}\right)$$

Where φ represents sediment porosity at the SWI and D_o_ is the molecular diffusion coefficient, and $\frac{dc}{dx}$ is the slope at the SWI. The D_o_ of citrate (3.22 × 10^−6^ cm^2^ s^−1^) was used due to similarities in composition and molecular weight to B_1_. The model ignores advection as is standard in similar sedimentation rate environments lacking bioturbation (e.g., Hammond et al., 1996).

## Results

### Pore water profiles

**Figure 2:**
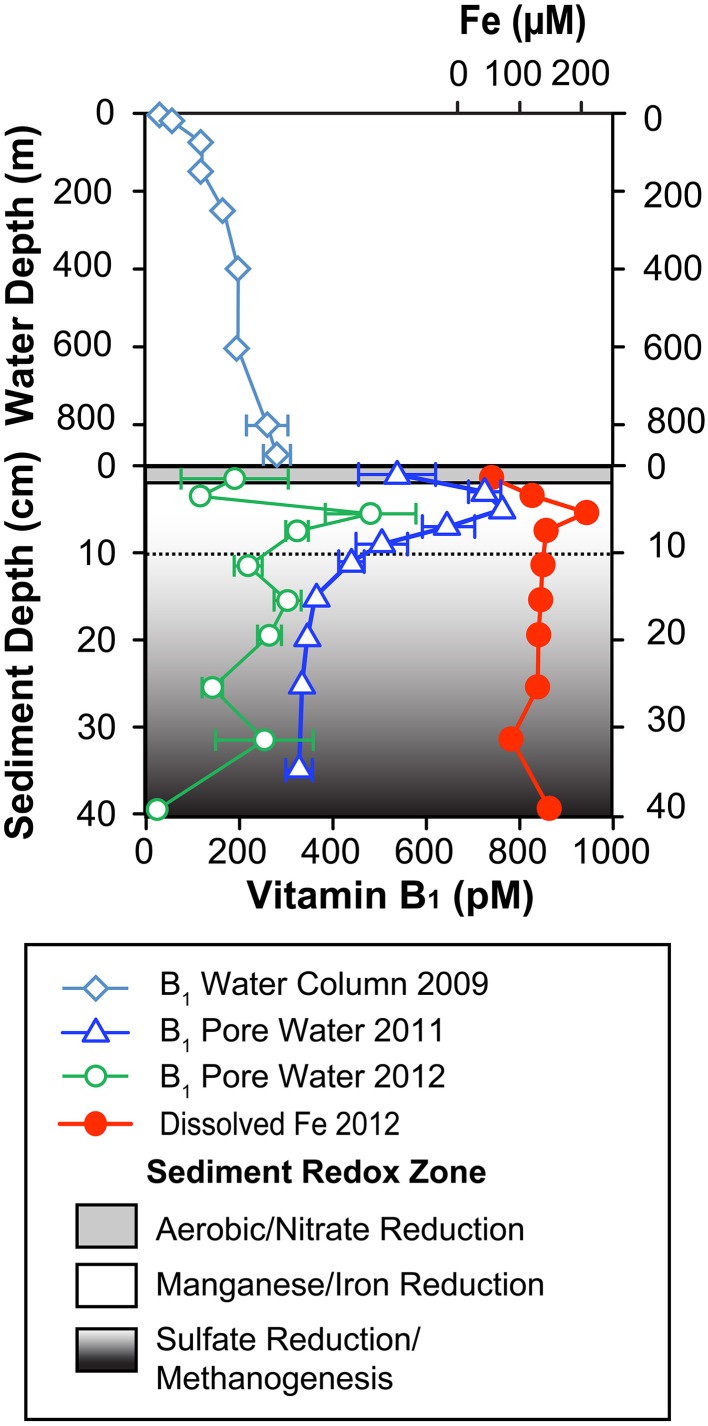
**B_1_ water column and pore water concentrations collected from Santa Monica Basin**. The upper portion of the profiles represents water column B_1_ concentrations collected in October 2009 and originally presented in Sañudo-Wilhelmy et al. (2012). The lower panel represents pore water concentrations of B_1_ collected in 2011 and 2012 as well as dissolved iron collected from a separate core in 2012. B_1_ error bars represent standard deviations of triple injections of a single sample. Shaded regions denote expected geochemical zonation based on the dissolved iron profile as well as a literature review of previously measured geochemical parameters in this same basin (Jahnke, 1990; Berelson et al., 2005; Komada et al., 2013).

Vitamin B_1_ concentrations in sediment pore water showed a consistent depth-profile shape in both sampling years (Figure 2). Concentrations were higher than water column values in most sampling depths of 2012 and all depths of 2011. Pore water concentrations ranged from 330 to 770 pM in 2011 and 30–480 pM in 2012 as compared to water column concentrations of 30–280 pM previously reported by Sañudo-Wilhelmy et al. (2012) for the same station location (Supplementary Material Table S3). Additionally, the water column concentrations consistently increased with depth such that the deepest water column sample (890 m) had the highest B_1_ concentration, ~280 pM. In both pore water profiles, vitamin B_1_ exhibited consistent maximum concentrations at ~5 cm sediment depth and subsequently decreased with depth in both sampling years. The maximum concentrations of B_1_ at ~5 cm sediment depth coincided with a maximum of dissolved iron (Figure 2; Supplementary Material Table S4).

### Modeled flux

The well-defined convex-upward B_1_ profile in 2011 allowed a one dimensional diffusion-transport model, based on Fick's First Law, to be applied to the pore water concentrations (see Supplementary Figure S1). A simple quadratic curve fit evaluated the inflection point to be at ~9 cm. This model was then applied to the five pore water data points within the top 9 cm of sediment and the bottom water concentration was fixed at 280 pM based on the deepest water column value. The model produced a statistically significant model fit with a high chi-squared value (chisq = 160). The concentration gradient was evaluated at the SWI and a potential B_1_ flux of 0.7 nmol m^−2^ d^−1^ was calculated out of the sediment. The less smooth shape of the 2012 profile (Figure 2) especially just below the sediment water interface, due to possible disturbance during transport and sampling or simply spatial variability, did not allow a good model fit for this sampling year. This is not uncommon for pore water sampling in deep marine sediments, for example of 8 total cores collected for DOC analysis in the same basin only 4 showed profiles consistent enough to allow a model fit (Komada et al., 2013).

### Potential algal growth yield

The calculated vitamin B_1_ flux described above is for the exchange across the SWI at depths of 900 m in a sedimentary basin, yet this represents the first flux estimate for any marine setting. Assuming our calculated flux is representative of similar fluxes occurring in shallow water environments, and that B_1_ degradation is minimal, a mass balance was applied to estimate the hypothetical growth response of such a sediment flux on a B_1_-limited surface ocean phytoplankton community. Using the estimated sediment flux (0.7 nmol m^−2^ d^−1^), a series of experimental phytoplankton cell growth yields ranging from to 2.2 × 10^−8^ to 3.6 × 10^−8^ pmol B_1_ cell^−1^ (Paerl et al., 2015), and a photic zone of ~20 m (Small et al., 1989), we estimated that this magnitude sediment flux could support an algal growth yield ranging from 9.8 × 10^5^ to 1.6 × 10^6^ cells L^−1^ d^−1^ (Supplementary Material Table S2).

## Discussion

### Vitamin B_1_ pore water profiles

The pore water depth profiles of B_1_ from two sampling years showed high maximum concentrations compared to previous field measurement as well as a consistent profile shape, especially considering the vitamin is present in such trace concentrations. This is in contrast to upper water column concentrations of B_1_ which can vary widely on fairly short time scales (months to days; e.g., Gobler et al., 2007; Koch et al., 2012) and do not necessarily show consistent profile shape (Sañudo-Wilhelmy et al., 2012). This is likely due to the stratified nature of deep marine sediments which result in predictable geochemical zones that are not as susceptible to mixing or large diurnal shifts in bacterio-phytoplankton activity which likely affect vitamin production and uptake in the surface ocean (Sañudo-Wilhelmy et al., 2014). The consistent B_1_ pore water concentration profile shape points to the existence of a stable mechanism for B_1_ release to the dissolved phase. Furthermore, the maximum dissolved pore water concentrations for B_1_ (770 pM) in 2011 were among the highest concentrations of any previously published values (see Table 1) almost 2 times higher than maximum water column concentrations. In fact the only other measurement that is within the same range was a single pore water value measured via HPLC (Okbamichael and Sañudo-Wilhelmy, 2005), supporting the hypothesis that sediments may represent universally elevated concentrations. Of the other water column measurements (Table 1), we note that some of the highest measurements are either found in shallow embayments likely affected by high benthic fluxes (Okbamichael and Sañudo-Wilhelmy, 2005) or anoxic marine basins such as SMB (Sañudo-Wilhelmy et al., 2012). These high concentrations suggest that vitamin production within the pore waters could be an important vitamin source for both the sediments and the water column. Additionally, of the 56 sediment bacteria and archaea surveyed in our genomic review, 74% of them were B_1_ prototrophs with all of the genes necessary to synthesize the vitamin *de novo* (see Supplementary Material Table S1).

**Table 1:** **Environmental marine measurements of vitamin B_1_**.

**Study area**	**Concentration range (pM)**	**References**
SMB pore water	30–770	This study
Marine pore water from Flax Pond, NY	750	Okbamichael and Sañudo-Wilhelmy, 2005
Shallow Embayments in Peconic River and Stony Brook Harbor, NY	230–310	Okbamichael and Sañudo-Wilhelmy, 2005
California-Baja Pacific Margin	<0.81–314	Sañudo-Wilhelmy et al., 2012
Western Tropical North Atlantic, Amazon River Plume	<0.81–230	Barada et al., 2013
Long Island Sound, NY	<10–220	Vishniac and Riley, 1961
Peconic River, NY	12–190	Gobler et al., 2007
Quantuck Bay, NY	7–169	Koch et al., 2013
Old Fort Pond, NY	0.1–112	Gobler et al., 2007; Koch et al., 2012
Long Island Sound, NY	<0.10–99	Koch et al., 2012
Scripps Institute of Oceanography Pier	20–40	Carlucci and Silbernagel, 1966

The shape of the B_1_ pore water profile showed similarity to our dissolved iron profile as both showed peaks at ~5 cm. Manganese, which has been measured in the same basin in other studies, also shows a coincident peak at ~5 cm depth (Jahnke, 1990). This implies that the largest B_1_ production was occurring within a geochemical zone of iron and manganese reduction as defined by the classic redox cascade of terminal electron acceptors (Figure 2; Froelich et al., 1979). We are unaware of any biological mechanism linking B_1_ to metal reduction, however many iron and manganese reducers are B_1_ prototrophs (see Supplementary Material Table S1). Future culture experiments on sediment isolates from this sediment zone may help to explain why the elevated pore water concentrations occurred at this depth.

Previous measurements of dissolved organic carbon (DOC) in this same basin also showed elevated concentrations starting around ~5 cm (Komada et al., 2013). As an organic molecule, B_1_ is part of the DOC pool, albeit a very small proportion (pM versus mM concentrations). Thus, processes driving changes in pore water DOC may also contribute to the profile shape of B_1_, namely organic carbon remineralization. B_1_ is a required cofactor for many important C-C breaking decarboxylase enzymes (Sañudo-Wilhelmy et al., 2014) which may serve as a possible mechanism linking the dissolved concentrations of DOC to B_1_. Future environmental sampling and culture experiments targeted at carbon remineralization and B_1_ production will be needed to confirm the validity of this proposed connection.

### Vitamin B_1_ benthic flux

The diffusive flux of 0.7 nmol m^−2^ d^−1^ out of the sediment represents the first such estimate ever made and therefore we lack a good comparison in order to judge the magnitude or significance of this flux. However, the algal growth yield calculation resulted in rates of cellular production (see Supplementary Material Table S2) some of which fall within the range for a phytoplankton bloom (Anderson et al., 2002). Certain caveats go along with these calculations, the most important being that we are explicitly not implying that the flux measured in SMB is reaching the surface waters. Instead, we assume that the calculated sediment flux may be representative of similar fluxes in more shallow environments as has been hypothesized in other studies (Okbamichael and Sañudo-Wilhelmy, 2005). Such a shallow water environment where a B_1_ sediment flux would be particularly relevant would include shallow embayments, marshes, lagoons, or other environments that experience significant mixing and/or deep seasonal upwelling in order to allow transport of B_1_-rich bottom water to surface waters. Furthermore, if the hypothesis that B_1_ is linked to DOC proves correct, we would expect that shallow sediments, which are generally more organic rich and host higher bacterial abundances, likely produce significantly higher B_1_ fluxes, and therefore the hypothetical growth yield can be considered a conservative estimate. Another assumption is that the surface algal community could be vitamin B_1_-limited, which is possible given that 20% of genomic surveyed algae and 27% of cultured phytoplankton are B_1_ auxotrophs (Croft et al., 2006; Sañudo-Wilhelmy et al., 2014), including many harmful algal bloom species (Tang et al., 2010). Of course additional variables would affect the initiation and response of the microbial community to the addition of B_1_ including potential degradation prior to biologic uptake, competitive auxotrophic consumption of B_1_, and additional release or transport during bloom die off (Sañudo-Wilhelmy et al., 2014). Despite these unknowns, our model suggests that benthic fluxes of B_1_ occur at physiologically relevant rates and could impact surface primary production under a vitamin-limited regime, which appears to exist in many regions of today's oceans (Bertrand and Allen, 2012; Sañudo-Wilhelmy et al., 2012).

## Summary and future directions

Here we presented the first deep-sea sediment pore water profiles of the universally required vitamin B_1_. Our data showed a stable profile, hinting at a yet-to-be-determined link to a fundamental metabolic sediment cycle. Additionally we showed that sediments might serve as a source of B_1_ to the water column. Future studies are needed in order to constrain spatial and temporal variability of sediment B_1_ fluxes. While we cannot provide an unequivocal link between B_1_ and microbial producers or consumers, these pore water profiles serve as a starting point to formulate future hypotheses. Interesting avenues for future studies include whether B_1_ can be limiting to sediment microbes in a similar way to their demonstrated limitation to surface water organisms despite the high pore water concentrations. Recent studies support the idea that B_1_ has the potential to act as an ectocrine intermediate and perhaps a limiting nutrient based on a recent finding of auxotrophy in the widespread and highly abundant phylum chloroflexi (Rodionova et al., 2014), the members of which represent a significant abundance of bacteria in some shallow and deep sediments based on genomic studies (e.g., Blazejak and Schippers, 2010; Jorgensen et al., 2012). Such hypotheses, when coupled with *in situ* prokaryotic diversity techniques, and physiological studies using bacterial isolates from the different biogeochemical zones, will elucidate the role of vitamin B_1_ on community function and composition both within the sediment and as a source to the water column.

### Conflict of interest statement

The authors declare that the research was conducted in the absence of any commercial or financial relationships that could be construed as a potential conflict of interest.
