# Supplementary material for: Vitamin B1 in marine sediments: pore water concentration gradient drives benthic flux with potential biological implications
Source: Front Microbiol. 2015 May 12;6:434. doi: 10.3389/fmicb.2015.00434 (PMC4428219; doi:10.3389/fmicb.2015.00434)
Supplement: Supplementary file 1 [file DataSheet1.DOCX]

***Supplementary Material***

**Vitamin B_1_ in marine sediments: pore water concentration gradient drives benthic flux with potential biological implications**

**Danielle Monteverde^1*^, Laura Gómez-Consarnau^2^, Lynda Cutter^2^, Lauren Chong^1^, William Berelson^1^ and Sergio A. Sañudo-Wilhelmy^1,2^**

^1^University of Southern California, Department of Earth Sciences, Los Angeles, CA, USA

^2^University of Southern California, Department of Biological Sciences, Los Angeles, CA, USA

*** Correspondence:** Danielle Monteverde, University of Southern California, Department of Earth Sciences, Zumberge Hall of Science (ZHS), 3651 Trousdale Pkwy, Los Angeles, CA 90089-0740, USA.

[dmonteve@usc.edu](mailto:dmonteve@usc.edu)

1. **Supplementary Data**
   1. **Genomic review**

A genomic review used for the discussion of our results was performed on whole-genome sequenced bacteria and archaea from marine sediments or marine soil, according to the annotations on the Joint Genome Institute’s Integrated Microbial Genomes and Metagenomes (IMG) database (<http://img.jgi.doe.gov>). The presence or absence of B-vitamin synthesis pathways was confirmed using Kegg metabolic maps (<http://www.genome.jp/kegg/pathway.html>) and RAST (<http://rast.nmpdr.org/>) for the genomes available on these additional databases. These genomes likely represent only a small fraction of the sediment microbial communities *in situ*, as most environmental bacteria have not been isolated in culture. However, our data indicates that both vitamin synthesizers and auxotrophs co-habit in marine sediments. Future studies will be needed to allow an accurate description of the microbes that dominate these activities.

The vitamin B_1_ synthesis pathway was considered present when genomes contained clusters of orthologous groups (COGs) for both *thiC* (COG0422) and *thiG* (COG2022) genes for bacteria, and the *thiC* (COG0422) and *thi4* (COG1635) genes for archaea.

**Supplementary Table S1. Survey of B-vitamin pathways present in cultured whole genome sequenced marine sediment organisms**

| **Organism** | **ThiC** | **ThiG/Thi4** | **B_1_** |
| --- | --- | --- | --- |
| Alcanivorax borkumensis SK2 | Yes | Yes | Yes |
| Alteromonas taeanensis SN2 | Yes | Yes | Yes |
| Bacillus sp. SG-1 | No | Yes | No |
| Beggiatoa sp. PS | Yes | Yes | Yes |
| Beggiatoa sp. SS | No | No | No |
| Caulobacter crescentus CB15 | Yes | Yes | Yes |
| Caulobacter crescentus NA1000 | Yes | Yes | Yes |
| Cyanobium sp. PCC 7001 | Yes | Yes | Yes |
| Cycloclasticus pugetii PS-1 | Yes | Yes | Yes |
| Cytophaga hutchinsonii ATCC 33406 | Yes | Yes | Yes |
| Dasania marina DSM 21967 | No | No | No |
| Delta proteobacterium NaphS2 | Yes | No | No |
| Desulfobacter curvatus DSM 3379 | Yes | Yes | Yes |
| Desulfobacter postgatei 2ac9 | Yes | Yes | Yes |
| Desulfobacterium autotrophicum HRM2, DSM 3382 | Yes | No | No |
| Desulfotalea psychrophila LSv54 | Yes | Yes | Yes |
| Erythrobacter sp, SD-21 | Yes | Yes | Yes |
| Ferrimonas balearica PAT, DSM 9799 | Yes | Yes | Yes |
| Flexithrix dorotheae DSM 6795 | Yes | Yes | Yes |
| Gamma proteobacterium sp. NOR51-B | No | No | No |
| Gamma proteobacterium sp. NOR5-3 | No | No | No |
| Geobacillus kaustophilus HTA426 | Yes | Yes | Yes |
| Halobacteroides halobius MD-1, DSM 5150 | Yes | Yes | Yes |
| Haloplasma contractile SSD-17B | No | No | No |
| Hippea maritima MH2, DSM 10411 | Yes | No | No |
| Kangiella aquimarina DSM 16071 | Yes | Yes | Yes |
| Kocuria rhizophila DC2201 | No | Yes | No |
| Kordiimonas gwangyangensis DSM 19435 | Yes | Yes | Yes |
| Magnetospirillum magnetotacticum MS-1 | Yes | Yes | Yes |
| Marinobacter manganoxydans MnI7-9 | Yes | Yes | Yes |
| Methanocaldococcus jannaschii DSM 2661 | Yes | Yes | Yes |
| Methanocaldococcus villosus KIN24-T80 | Yes | Yes | Yes |
| Methylomonas methanica MC09 | Yes | Yes | Yes |
| Muricauda ruestringensis B1, DSM 13258 | No | No | No |
| Nitrobacter sp. Nb-311A | Yes | Yes | Yes |
| Paenibacillus terrigena DSM 21567 | Yes | Yes | Yes |
| Psychrobacter sp. PRwf-1 | Yes | Yes | Yes |
| Pyrococcus furiosus COM1 | Yes | Yes | Yes |
| Salinisphaera shabanensis E1L3A | Yes | Yes | Yes |
| Salinispora arenicola CNS-205 | Yes | Yes | Yes |
| Salinispora pacifica DSM 45543 | Yes | Yes | Yes |
| Salinispora pacifica DSM 45544 | Yes | Yes | Yes |
| Salinispora pacifica DSM 45546 | Yes | Yes | Yes |
| Salinispora pacifica DSM 45547 | Yes | Yes | Yes |
| Salinispora pacifica DSM 45548 | Yes | Yes | Yes |
| Salinispora pacifica DSM 45549 | Yes | Yes | Yes |
| Sciscionella marina DSM 45152 | Yes | Yes | Yes |
| Shewanella halifaxensis HAW-EB4 | Yes | Yes | Yes |
| Shewanella piezotolerans WP3 | Yes | Yes | Yes |
| Shewanella sediminis HAW-EB3 | Yes | Yes | Yes |
| Shewanella sp. W3-18-1 | Yes | Yes | Yes |
| Shewanella violacea DSS12 | Yes | Yes | Yes |
| Staphylothermus hellenicus P8, DSM 12710 | No | Yes | No |
| Sulfolobus islandicus L.D.8.5 | No | Yes | No |
| Sulfolobus tokodaii 7, JCM 10545 | Yes | Yes | Yes |
| Sulfurimonas autotrophica OK10, DSM 16294 | Yes | Yes | Yes |
| Thermococcus gammatolerans EJ3 | Yes | Yes | Yes |
| Thermoplasma volcanium GSS1 | No | No | No |
| Thermosediminibacter oceani JW/IW-1228P, DSM 16646 | No | No | No |
| Thermotoga maritima MSB8 | Yes | No | No |
| Thermotoga maritima MSB8, DSM 3109 | Yes | No | No |
| Thiobacillus denitrificans DSM 12475 | Yes | Yes | Yes |
| Verrucosispora maris AB-18-032 | Yes | Yes | Yes |
| Vulcanisaeta distributa DSM 14429 | Yes | Yes | Yes |
| Zunongwangia profunda SM-A87 | Yes | Yes | Yes |

- 1. **Potential algal growth yield calculation**

**Supplementary Table S2. Potential Benthic-Sourced B_1_ Cellular Production**

| **Organism** | **Cellular Quotas^a^** | **Cellular production** |
| --- | --- | --- |
|  | **(pmol B_1_/cell)** | **(cells/L/d)** |
| O. lucimarinus CCE9901 | 2.20E-08 | 1.59E+06 |
| O. lucimarinus CCE9901 (L:D) | 2.72E-08 | 1.29E+06 |
| Ostreococcus sp. CCE1301 | 3.58E-08 | 9.78E+05 |
| M. pusilla CCMP487 | 2.55E-08 | 1.37E+06 |
| ^a^Paerl et. al (2015) | | |

Cell production rates were calculated using previously published experimental minimum vitamin quotas determined from laboratory culture experiments. These quotas were converted into cellular production rates by utilizing the modelled diffusive benthic flux of B_1_ (700 pmol m^-2^ d^-1^) and assuming a photic zone of 20 meters (Small et al., 1989) to reach cellular production in units of cells L^-1^ day^-1^.

- 1. **Raw data**

**Supplementary Table S3. Dissolved B_1_ Concentrations in SMB Marine Pore Waters**

|  | **2011** |  |  | **2012** |  |
| --- | --- | --- | --- | --- | --- |
| **Depth** | **Avg** | **Stdev** | **Depth** | **Avg** | **Stdev** |
| **(cm)** | **(pM)** | **(pM)** | **(cm)** | **(pM)** | **(pM)** |
| 1 | 539.63 | 82.47 | 1.5 | 180.53 | 114.87 |
| 3 | 727.14 | 34.63 | 3.5 | 107.04 | 15.62 |
| 5 | 764.82 | 4.92 | 5.5 | 471.59 | 97.08 |
| 7 | 649.76 | 56.08 | 7.5 | 314.20 | 24.44 |
| 9 | 507.10 | 55.23 | 11.5 | 209.51 | 29.97 |
| 11 | 441.84 | 27.21 | 15.5 | 293.89 | 28.93 |
| 15 | 364.08 | 9.95 | 19.5 | 255.15 | 25.74 |
| 20 | 347.00 | 16.71 | 25.5 | 132.69 | 21.76 |
| 25 | 336.17 | 18.82 | 31.5 | 244.32 | 104.71 |
| 35 | 329.72 | 28.52 | 39.5 | 15.14 | 11.74 |
| *Average and standard deviation values are calculated from triple injections of a single sample | | | | | |

**Supplementary Table S4. 2012 Dissolved Fe Concentrations in SMB Marine Pore Waters**

| **Sediment Depth** | **Fe** |
| --- | --- |
| **(cm)** | **(µM)** |
| 1 | 55 |
| 3 | 120 |
| 5 | 208 |
| 7 | 143 |
| 11 | 137 |
| 15 | 134 |
| 19 | 131 |
| 25 | 129 |
| 31 | 87 |
| 39 | 148 |

1. **References**

Paerl, R.W., Bertrand, E.M., Allen, A.E., Palenik, B., and Azam, F. (2015). Vitamin B1 ecophysiology of marine picoeukaryotic algae: Strain-specific differences and a new role for bacteria in vitamin cycling. *Limnology and Oceanography***,** n/a-n/a. doi: 10.1002/lno.10009.

Small, L.F., Landry, M.R., Eppley, R.W., Azam, F., and Carlucci, A.F. (1989). Role of plankton in the carbon and nitrogen budgets of Santa-Monica-Basin, California. *Marine Ecology Progress Series* 56**,** 57-74. doi: 10.3354/meps056057.

## Supplementary Figures

**Supplementary Figure S1.** **One dimensional diffusion-reaction model applied to the B_1_ pore water profile.** Triangles represent sampled concentrations and line (red and green) represents separate zones of model fit (chisq=160).
