# Supplementary figures and images for: Vitamin B1 in marine sediments: pore water concentration gradient drives benthic flux with potential biological implications
Source: Front Microbiol. 2015 May 12;6:434. doi: 10.3389/fmicb.2015.00434 (PMC4428219; doi:10.3389/fmicb.2015.00434)

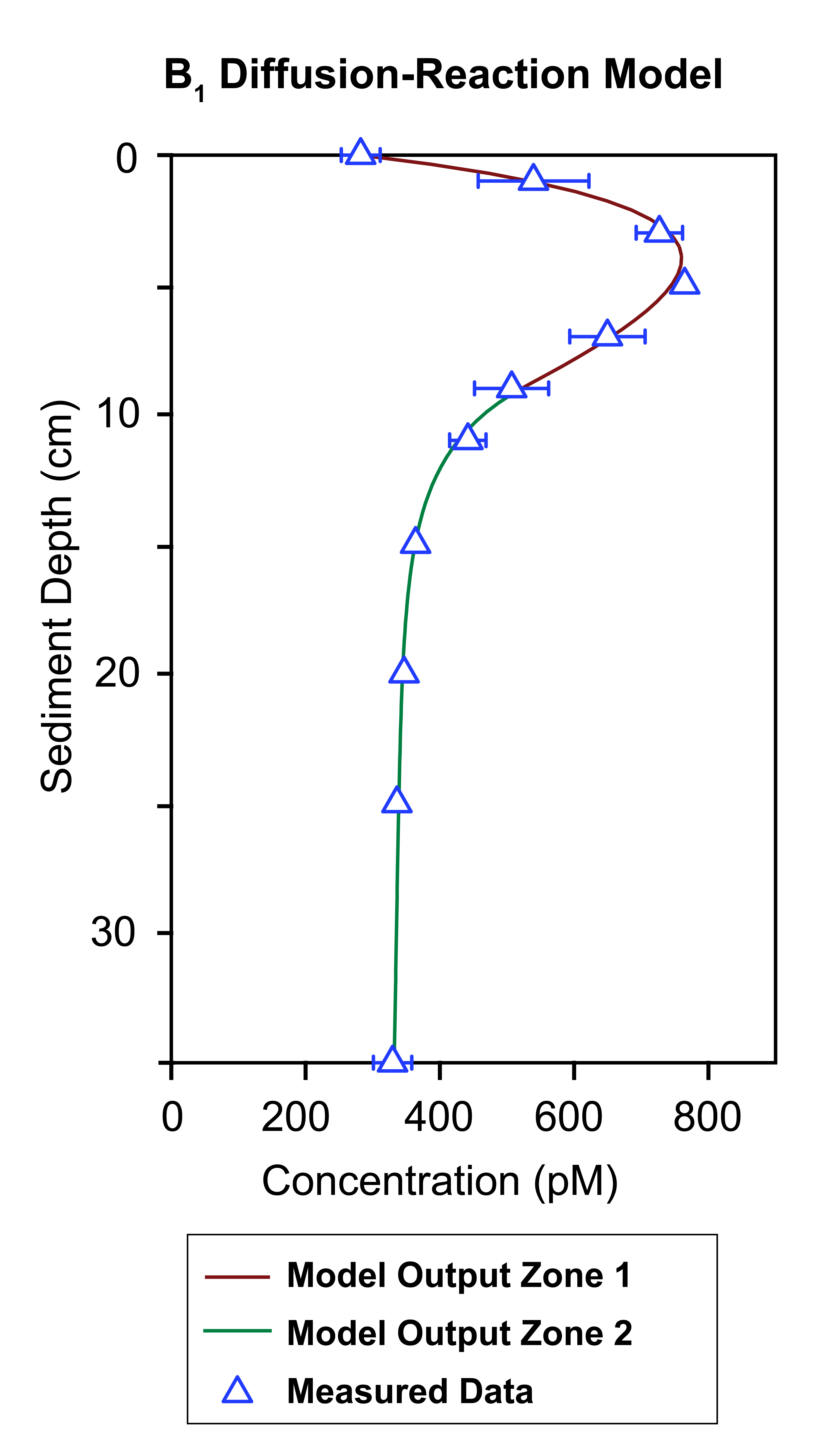

Supplement: Supplementary file 2 [file Image1.JPEG]
